# Fully Solution-Processable Fabrication of Multi-Layered Circuits on a Flexible Substrate Using Laser Processing

**DOI:** 10.3390/ma11020268

**Published:** 2018-02-09

**Authors:** Seok Young Ji, Wonsuk Choi, Hoon-Young Kim, Jin-Woo Jeon, Sung-Hak Cho, Won Seok Chang

**Affiliations:** 1 Department of Nano Mechanics, Nanomechanical Systems Research Division, Korea Institute of Machinery and Materials (KIMM), 156 Gajeongbuk-Ro, Yuseong-Gu, Daejeon 34103, Korea; ji10047@kimm.re.kr; 2 Department of Nano-Mechatronics, Korea University of Science and Technology (UST), 217 Gajeong-Ro, Yuseong-Gu, Daejeon 34113, Korea; cws@kimm.re.kr (W.C.); hykim@kimm.re.kr (H.-Y.K.); shcho@kimm.re.kr (S.-H.C.); 3 Department of Laser & Electron Beam Application, Korea Institute of Machinery and Material (KIMM), 156 Gajeongbuk-Ro, Yuseong-Gu, Daejeon 34103, Korea; jwj@kimm.re.kr

**Keywords:** interconnection, multi-layer patterning, laser sintering, femtosecond laser ablation

## Abstract

The development of printing technologies has enabled the realization of electric circuit fabrication on a flexible substrate. However, the current technique remains restricted to single-layer patterning. In this paper, we demonstrate a fully solution-processable patterning approach for multi-layer circuits using a combined method of laser sintering and ablation. Selective laser sintering of silver (Ag) nanoparticle-based ink is applied to make conductive patterns on a heat-sensitive substrate and insulating layer. The laser beam path and irradiation fluence are controlled to create circuit patterns for flexible electronics. Microvia drilling using femtosecond laser through the polyvinylphenol-film insulating layer by laser ablation, as well as sequential coating of Ag ink and laser sintering, achieves an interlayer interconnection between multi-layer circuits. The dimension of microvia is determined by a sophisticated adjustment of the laser focal position and intensity. Based on these methods, a flexible electronic circuit with chip-size-package light-emitting diodes was successfully fabricated and demonstrated to have functional operations.

## 1. Introduction

Functional-ink-based printing technology has greatly advanced in recent years for use with flexible electronics [1,2]. Printing techniques, such as micro-contact (μ-contact) [2,3,4], inkjet [5,6,7,8], screen [9,10], flexography [11,12] and gravure [13,14,15] approaches, have made it possible to fabricate conductive patterns in large areas at a reasonable cost. As printing technologies have matured, electronic applications, such as resistor–capacitor circuits [16], radio frequency identification [17], sensors [18], displays [19], organic field-effect transistors [20,21], and solar cells [22], have been reported. However, these techniques support only single-layer circuit printing and are thus not well-suited for the manufacturing of multi-layer circuits. Multi-layer circuit manufacturing is desirable because it not only reduces the circuit routing complexity, but it also enables the fabrication of integrated circuits (IC) [23,24,25,26]. Moreover, multi-layer circuit fabrication requires the stacking of single-layer circuits and hole creation for an interconnecting layer-to-layer structure. Previous studies suggested an interconnection method by dry etching [23,24], which is slow, expensive, and harmful to the environment. Another approach is to use a drilling mechanism that employs a hole opener [26]. This method consumes a small amount of energy and enables interconnection in a short time. Nevertheless, it is only available for the fabrication of double-sided circuits, and it is difficult to implement the miniaturization of the electronic device because the minimum via-hole size is 500 μm.

In this study, we developed a fully solution-processable fabrication process for a multi-layer circuit on a flexible substrate using a combined method of selective laser sintering (SLS) and ablation (SLA). SLS with double irradiation enables low-temperature metal patterning without damaging the heat-sensitive substrate [27]. The double irradiation method uses a higher laser power than the single irradiation method to fabricate electrodes with high conductivity on heat-sensitive substrates. In addition, we introduced femtosecond laser-based SLA through a polyvinylphenol (PVP) insulator to create microvias for interconnections between layers. For ablating insulating layers without damaging the bottom electrode, we investigated the correlation between a femtosecond laser and materials such as the sintered Ag electrode and the PVP insulating layer to remove only the PVP insulating layer. We experimentally measured the ablation threshold of the sintered Ag electrode (E_Ag_) and PVP insulating layer (E_PVP_) based on a Liu-plot. As a result, we were able to ablate the PVP insulating layer without damaging the bottom circuit and polymer substrate. The laser-based process was managed by the optimized conditions of the laser fluence and focal position to achieve a suitable microvia diameter and depth. A multi-layered flexible electric circuit was constructed using the suggested process and operated with an assembly of chip-size-package light-emitting diodes (LEDs) for process verification.

## 2. Materials and Methods

### 2.1. Experimental Setup

A solid state Nd:YAG continuous wave (CW) laser (mpc 6000, Ventus 532, Laser Quantum, Germany) with a wavelength of 532 nm was implemented for the SLS, which has a maximum power of 1.6 W, power stability of <0.4% root-mean-square (rms), and beam size of 1.5 ± 0.1 mm. A galvano scanner (intelliSCAN^®^10, SCANLAB, Puchheim, Germany) was used for the laser beam scanning. It has a maximum making speed of 3.0 m/s, scan area of 45 × 45 mm^2^, scan angle of ±22 degrees, and nonlinearity of <3.5 mrad. The SLA source was the second harmonic 515-nm femtosecond Yb:KGW laser from Light Conversion (Pharos SP, Light Conversion, Vilnius, Lithuania), which has a maximum power of 6 W, pulse duration of 190 fs, maximum pulse energy of >1.0 mJ, and beam quality of TEM00; M2 < 1.3. The laser beams were focused at a normal incidence onto the insulating layer by a single ×50 infinity corrected objective lens with a numerical aperture (NA) of 0.42.

### 2.2. Materials and Preparation

For this study, a commercially available conductive ink and insulating layer were applied. The silver (Ag) nanoparticle (NP) inks were from Harima Chemicals, Inc. (NPS-J) in Tyoko, Japan, the capital of Japan and had Ag particles with a 12-nm mean diameter and a metal content of 65%. The poly (vinylphenol) (PVP) and methylated poly (melamine-*co*-formaldehyde) (MMF) for the polymer insulator layer were purchased from Sigma-Aldrich and used without further purification. The PVP-MMF solution (PVP:MMF = 1:1.25) was dissolved in propylene glycol monomethyl ether acetate (PGMEA) at a solid concentration of 100 mg/mL. These solutions were then filtered through a 0.45-μm polytetrafluoroethylene syringe filter before deposition by spin-coating.

### 2.3. Multi-Layer Patterning Process

**Figure 1:**
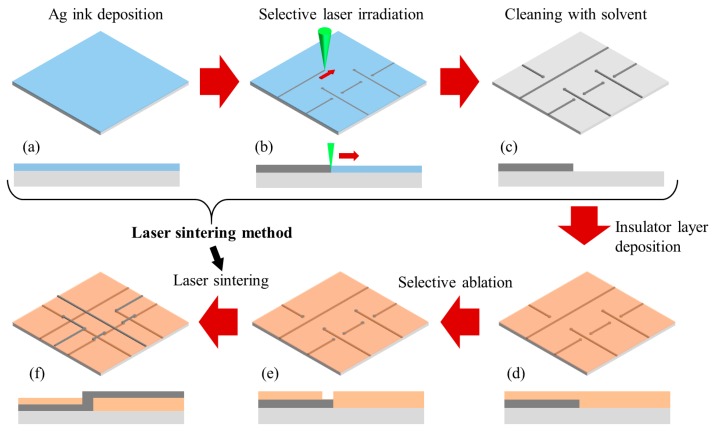
Schematic of the multi-layer patterning process using selective laser sintering and ablation.

Figure 1 shows a detailed multi-layer patterning process flow. First, the bottom pattern was prepared by SLS on a polyimide (PI) substrate, as shown in Figure 1a–c. The Ag NP ink was spin-coated onto the PI substrate, followed by pre-drying in ambient conditions on a hot plate at 70 °C to stop the flow of the Ag NP ink (Figure 1a). The thickness of the Ag NP ink films before sintering was ~70 nm. The prepared Ag NP film was selectively sintered by a scanning laser beam with a Galvano scanner to draw the desired patterns (Figure 1b). Then, the un-sintered Ag NPs were simply washed away with the solvent (toluene) to reveal the metal patterns (Figure 1c).

To remove the remaining impurities and increase the surface energy, the substrates with patterns were sequentially cleaned in an ultrasonic bath with deionized water and 2-propanol for 10 min each. The PVP solution was spin-coated onto the cleaned substrates, followed by pre-baking at 120 °C for 10 min and inducing thermal cross-linking at 220 °C for 60 min (Figure 1d). The thickness of the PVP polymer insulator films was ~500 nm. Next, the insulator film was selectively ablated using the femtosecond laser (Figure 1e). The laser sintering method was sequentially applied to form the top patterns and interconnection between the top and bottom circuit (Figure 1f).

## 3. Results

**Figure 2:**
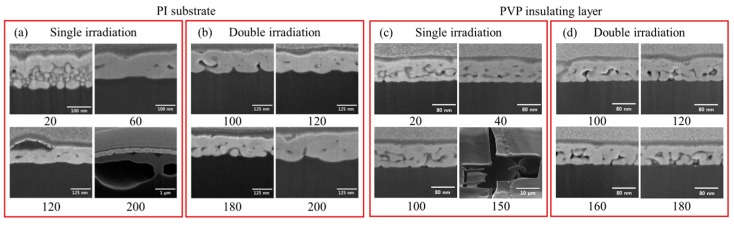
Cross-sectional SEM images of Ag electrode sintered by (**a**,**c**) single and (**b**,**d**) double laser irradiation with different power on PI substrate and PVP insulating layer. The numbers underneath each SEM image indicate the laser power.

To fabricate the multi-layer circuit, conductive patterns and the interconnection between layers must be achieved on the heat-sensitive substrate without thermal damage. Figure 2 shows cross-sectional scanning electron microscope (SEM) (Nova 200, NanoLab, Milpitas, CA, USA) images of patterns using laser sintering at various laser powers using the single irradiation method (Figure 2a,c) and double irradiation method (Figure 2b,d) on the PI substrate and PVP insulating layer. The Ag NPs show a high-energy absorption rate at a wavelength of 532 nm [28], which enables surface sintering features even at a low laser power of 10 mW. The patterns fabricated with a laser power of 40 mW or lower show surface sintering; however, the interior ink of the pattern is not completely sintered. Thus, it was detached or damaged during the cleaning process due to insufficient adhesion of the partially sintered Ag layer to the substrate.

A higher laser power should give a higher electric conductivity and adhesion of sintered Ag NP film compared to lower ones because of the incomplete coalescence of Ag nanoparticles and the diminished electron scattering [28,29,30,31]. However, when we induced a high laser power to obtain high electric conductivity, portions of the patterns incurred defects and burning effects because the higher laser power directly irradiated the heat-sensitive substrate through the Ag NP layer. It thus influenced the pattern conditions, such as conductivity and roughness. To circumvent this problem, we used a double laser sintering method, which involved two processes: first, irradiation using a low laser power was performed on Ag NP films to pre-sinter the surface layer, called surface-sintering (SS); second, the un-sintered interior ink was post-sintered by the high laser power, called complete-sintering (CS). 

In the SS step, a low (less than 20 mW) laser power successfully sintered the surface layer, which preserved the heat-sensitive substrate by the following high-power laser irradiation. When we irradiated with the high-power laser in the CS step, the interior Ag NP layer of the pattern was sintered by vertical heat conduction. The substrates remained unaffected by the direct laser irradiation on account of the surface layer sintered in the previous SS step.

Figure 2b shows optical images of the patterns using the double irradiation method. For this method, we employed 20 mW of laser power in the SS step and varied the laser power in the CS step. It was confirmed that the pattern was fabricated without damage in the conditions with the high laser power, which usually caused defects in the single irradiation method, as shown in Figure 2a,c. Nevertheless, some patterns incurred defects owing to the excessive vertical heat conduction, which affected the substrate in the CS step (laser power of 250 mW and 200 mW on the PI substrate and PVP insulating layer, respectively (Figures S1b and S2b).

To verify the patterns condition when irradiating with various laser powers, we measured the conductivity for the three process conditions: single laser irradiation, double irradiation with pre-sintering at a laser power of 10, and the latter approach at a laser power of 20 mW. The laser power was adjusted from 60 mW to 250 mW because the pattern fabricated with less than 40 mW was partially detached during the cleaning process. We attempted an experiment on the PI substrate (Figure S3a) and the PVP insulating layer (Figure S3b), respectively. The graphs of laser-power versus conductivity show that the measured conductivity increased as the laser power increased. The trend of increasing conductivity with laser power is readily elucidated by various sintering models [28,29,30,31]. It is noted that the Ag NP grain-growth mechanisms upon laser irradiation [28] can explain the high conductivity at a high laser-power; nonetheless, thermal damage was incurred by the printed pattern. The fabricated pattern with SS at 20 mW and CS at 240 mW had the highest conductivity, but it has been confirmed that substrate is damaged by 50% probability. We selected a laser power that did not damage the substrate, even after a test of 100 repetitions.

Consequentially, we fabricated the pattern with a conductivity of 6.21 × 10^5^ S/cm and an rms roughness of 7.09 ± 0.5 nm on PI without damage, using the double irradiation method with SS at a laser power of 20 mW and CS at 160 mW. This result was approximate to the conductivity of bulk silver of 6.3 × 10^5^ S/cm. On the other hand, the pattern on the PVP layer had slightly lower conductivity of 4.25 × 10^5^ S/cm. This is because we induced relatively low laser powers of 20 mW and 140 mW for SS and CS, respectively, considering the PVP layer thickness of 500 nm. We performed two experiments to check the robustness of the fabricated pattern. For the bias test, a pattern with a line width of 50 μm and a length of 20 mm was fabricated and maintained at a voltage of 50 V (compliance of maximum current was 100 mA) for more than 10 h, but the pattern was not damaged or broken. Also, after connecting the LED to the fabricated pattern, it was confirmed that the LED operated normally until the bending test was performed 100 times.

**Figure 3:**
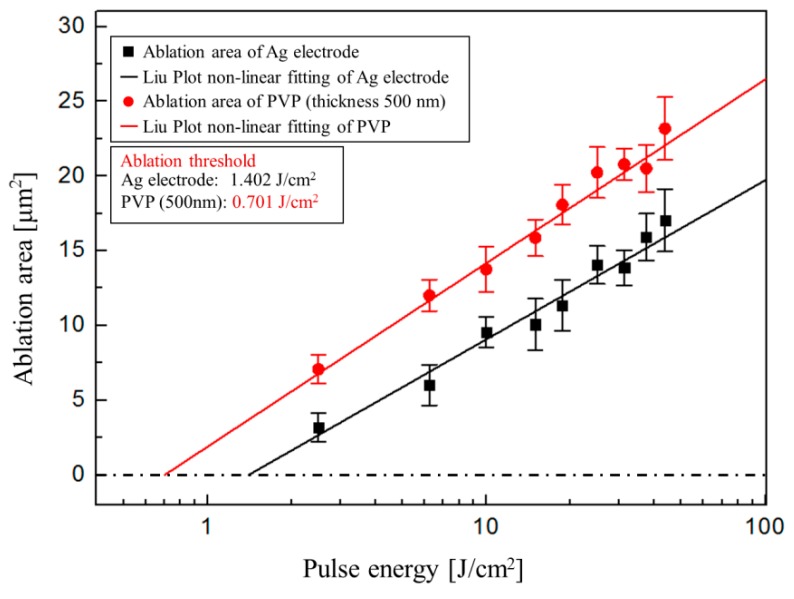
Liu-plot of ablation areas for laser irradiation on sintered Ag electrode and PVP insulating layer. The value of the ablation threshold has been extracted from experimental results. (Liu-plot non-linear fitting).

Multi-layer circuit manufacturing includes not only the fabrication of conductive patterns on a heat-sensitive substrate, but also an interconnection between layers. Thus, selective laser ablation using femtosecond laser was performed to make a microscale via-hole (named “microvia”) through the insulating PVP layer without damaging the bottom electrode. Generally, ultra-short pulse lasers such as femtosecond and picosecond lasers cause small thermal defects in the materials compared to long pulse laser (nanosecond pulse laser) irradiation [32]. Above all, femtosecond lasers can have high peak intensities with low pulse fluence via the ultrashort pulse durations, resulting in precise ablation with low heat-affected area. Using this attractive characteristic of femtosecond lasers, we used low laser fluence to ablate only PVP insulating layers in the interconnection region without damaging the bottom Ag electrode. To do this, we need to know the ablation threshold of the sintered Ag electrode (E_Ag_) and the PVP insulating layer (E_PVP_). The laser fluence would be determined between E_PVP_ and E_Ag_. To determine the ablation thresholds of each material for the complete series of the ablation spot, the relevant ablation areas have to be plotted versus laser pulse fluence on a logarithmic scale. Then the ablation threshold of each material can be derived by extrapolating the relationship between the laser fluence and the relevant areas on a Liu-plot [33,34]. For this purpose, the objective lens was focused on the surface of the materials, and the relevant ablation area was observed and measured with an optical microscope (OM), while increasing or decreasing laser fluence. The results of this analysis are shown in Figure 3. It is obvious that the value of E_PVP_ (0.701 J/cm^2^) for ablating the insulating layer is about half of E_Ag_ (1.402 J/cm^2^). Based on the results, we can fabricate microvia that can be used for interconnection by selected laser ablation with fluence (~0.9 J/cm^2^). The microvia diameter ranged from 100 μm to 10 μm, and the stable minimum diameter was 10 μm. After fabricating the microvia, the Ag NP ink filled the empty microvia space during the spin-coating step of the SLS process. In the laser-sintering step of the top pattern, the laser beam path was specifically modified for the double CS step on the microvia position to increase the heat penetration depth. 

**Figure 4:**
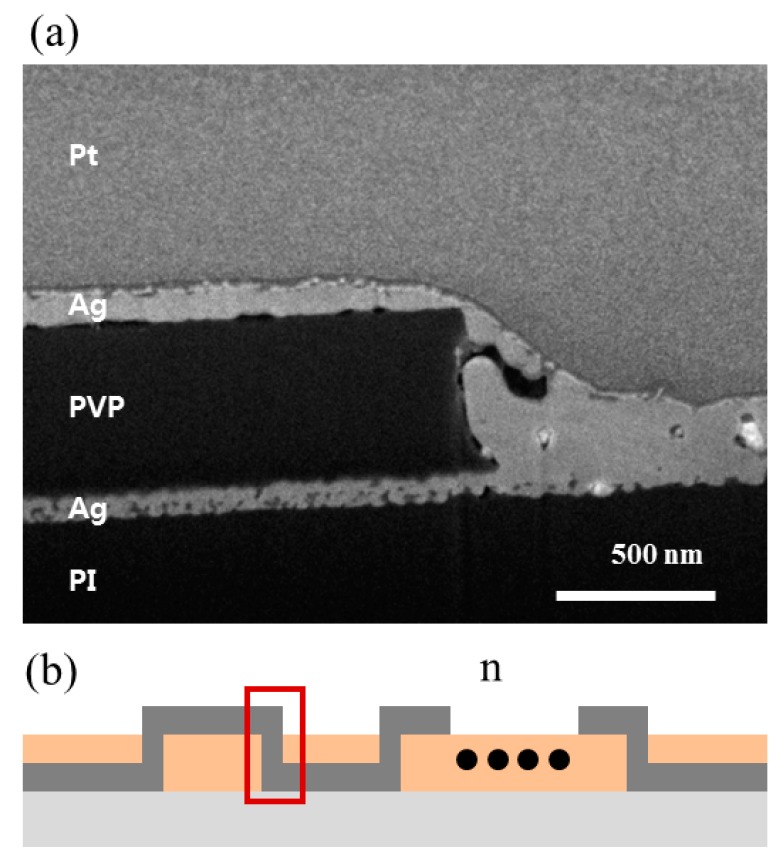
(**a**) Cross-sectional SEM image of the interconnected region between the top and bottom Ag electrodes; (**b**) schematic of cross-sectional sample structure fabricated by selective laser sintering (SLS) and selective laser ablation (SLA).

Figure 4a shows a cross-sectional scanning electron microscopy (SEM) image of the interconnected part between the top and bottom layers. To verify whether the interconnection affects the patterns, we measured the resistance of the patterns with a different number of interconnections, as shown in Figure 4b. Table 1 shows that the resistance has similar values regardless of the number of interconnections. Thus, we could expect that the electric current flow was not affected by the interconnection. Using the multi-layer patterning process with interconnections, more complicated circuits with two or more layers could be fabricated.

**Figure 5:**
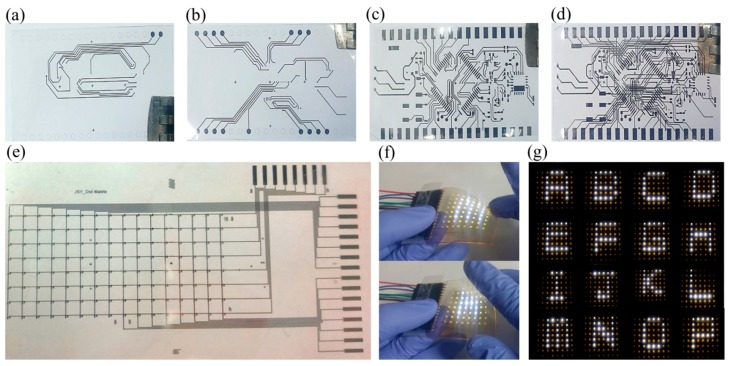
Optical photographs of micro-controller-unit (MCU) board circuit for (**a**) ground (first layer); (**b**) voltage (second layer); (**c**) main (third layer); and (**d**) final circuits fabricated by the multi-layer patterning process on the PI substrate; (**e**) Fabricated dot-matrix circuit using the multi-layer patterning process; (**f**) bending test of the fabricated electric device; (**g**) expression of various capital letters using light emitting diodes (LEDs).

We designed a flexible micro-controller-unit (MCU) circuit with triple layers consisting of a ground circuit on the first layer, a voltage circuit on the second layer, and a main circuit on the third layer. The device circuit has full size of 25 × 45 mm^2^ with a line width of 30 μm. The separated patterns of the ground, voltage, and main circuit on the PI are presented in Figure 5a–c, respectively. To achieve a multi-layered circuit on the same substrate, it usually requires a complex etching and deposition process for connecting between layers. We demonstrated that the entire MCU circuit could be fabricated by the multi-layer patterning process on the PI, as shown in Figure 5d.

Finally, we attempted to manufacture a functional device using the multi-layer patterning process to verify that this process is applicable to flexible applications. As shown in Figure 5e, the 8 × 16 dot matrix circuit was obtained with a full device size of 45 × 85 mm^2^, and an LED connection pad of 150 × 80 μm^2^. After patterning the dot matrix circuits, the chip-size-package LEDs were mounted on the prepared circuit. An MCU (mpc 430, Texas Instruments, Dallas, TX, USA), connected using copper wires (diameter ~500 μm), was used to input the signal to the LEDs. As shown in Figure 5f, the LED operation according to the input signal was well maintained during the bending state, thus demonstrating excellent possibilities for flexible electronic device applications. Figure 5g shows the various capital letters (from A to P) represented by the controlled input pins on the dot matrix.

## 4. Conclusions

Using a combined SLS and SLA method, we developed a fully solution-processable fabrication process for multi-layer patterns on a flexible substrate. We verified that the electric resistance of the fabricated patterns was not affected by the number of interconnections. Moreover, to demonstrate that the multi-layer patterning process can be used for flexible applications, an electric device circuit with two and three layers was fabricated using this process.

This research provides several guidelines for the study of the laser sintering method on a heat-sensitive substrate and the fabrication of multi-layer patterns. In addition, it enables the achievement of a desirable microvia diameter and suitable depth by controlling the laser focus position and intensity to make interconnections. The electric device fabricated with our technology showed successful operation. We expect that the proposed approach will be a key technology for implementing user-designed flexible electronic devices in the near future.

## Figures and Tables

**Table 1:** The measured resistances (mean ± standard deviation) according to the number of interconnections.

The Number of Interconnections	Resistance (Ω)
None	12.35 ± 0.33
1	12.37 ± 0.42
2	12.41 ± 0.35
3	12.40 ± 0.38
4	12.43 ± 0.36

## Supplementary Materials

The following are available online at http://www.mdpi.com/1996-1944/11/2/268/s1. Figure S1: Cross-sectional SEM image of electrode lines using (a) single irradiation method and (b) double irradiation method with the surface sintering of laser power of 20 mW at various laser powers. All printed patterns were fabricated on PI substrate. Figure S2: Cross-sectional SEM image of electrode lines using (a) single irradiation method and (b) double irradiation method with the surface sintering of laser power of 20 mW at various laser powers. All printed patterns were fabricated on PVP insulating layer. Figure S3: Electric conductivity versus laser power on (a) PI substrate and (b) PVP insulating layer.
