# Supplementary material for: Fully Solution-Processable Fabrication of Multi-Layered Circuits on a Flexible Substrate Using Laser Processing
Source: Materials (Basel). 2018 Feb 9;11(2):268. doi: 10.3390/ma11020268 (PMC5848965; doi:10.3390/ma11020268)
Supplement: Supplementary file 1 [file materials-11-00268-s001.pdf]

materials

Supplementary Information

Fully solution-processable fabrication of multi-layered circuits on flexible substrate using laser

Seok Young Ji[1, 2], Wonsuk Choi[2, 3], Hoon-Young Kim[2, 3], Jin-Woo Jeon[3], Sung-Hak Cho[2, 3] and Won Seok Chang[1, 2,*]

[1] Department of Nano Mechanics, Nanomechanical Systems Research Division, Korea Institute of Machinery and Materials (KIMM), 156 Gajeongbuk-Ro, Yuseong-Gu, Daejeon 34103, Korea; ji10047@kimm.re.kr
[2] Department of Nano-Mechatronics, Korea University of Science and Technology (UST), 217 Gajeong-Ro, Yuseong-Gu, Daejeon 34113, Korea; cws@kimm.re.kr (W.C.); hykim@kimm.re.kr (H.-Y.K.); shcho@kimm.re.kr (S.-H.C.)
[3] Department of Laser & Electron Beam Application, Korea Institute of Machinery and Material (KIMM), 156 Gajeongbuk-Ro, Yuseong-Gu, Daejeon 34103, Korea; jwj@kimm.re.kr
* Correspondence: paul@kimm.re.kr; Tel.: +82-42-868-7134

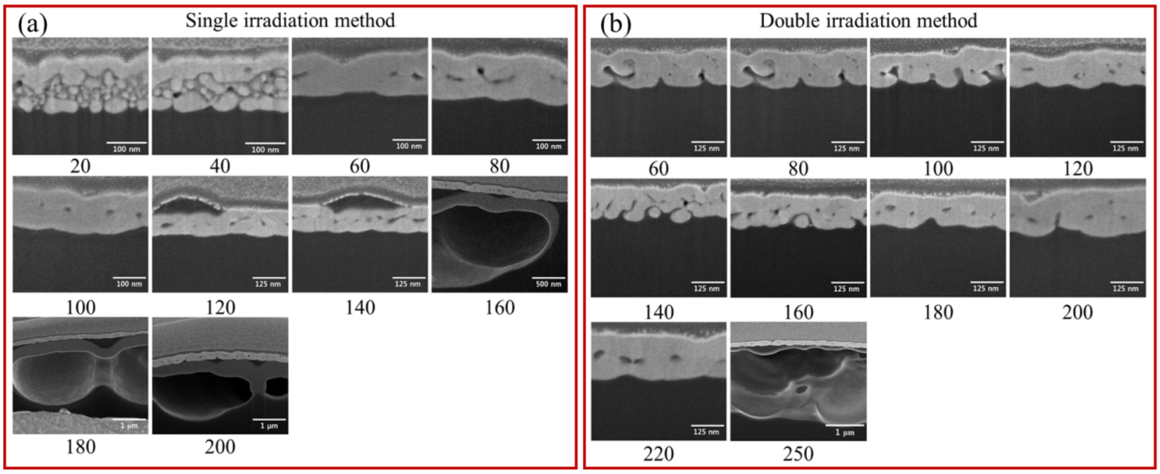

Figure S1. Cross-sectional SEM image of electrode lines using (a) single irradiation method and (b) double irradiation method with the surface sintering of laser power of 20 mW at various laser powers. All printed patterns were fabricated on PI substrate.

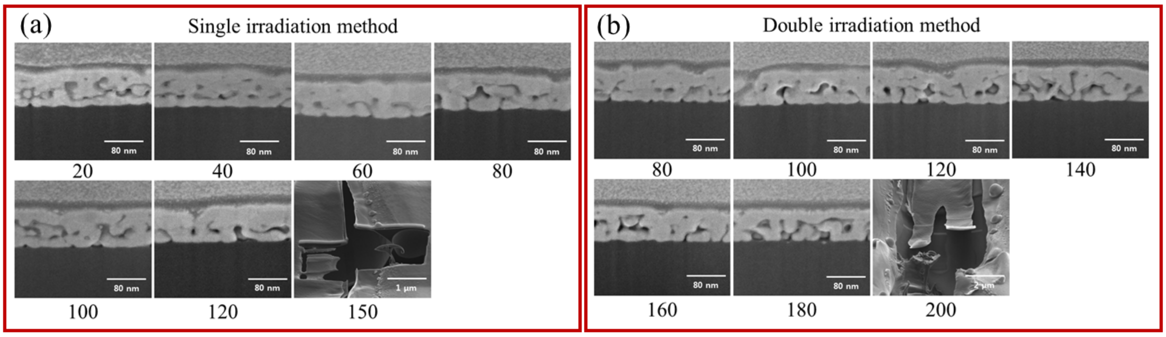

Figure S2. Cross-sectional SEM image of electrode lines using (a) single irradiation method and (b) double irradiation method with the surface sintering of laser power of 20 mW at various laser powers. All printed patterns were fabricated on PVP insulating layer.

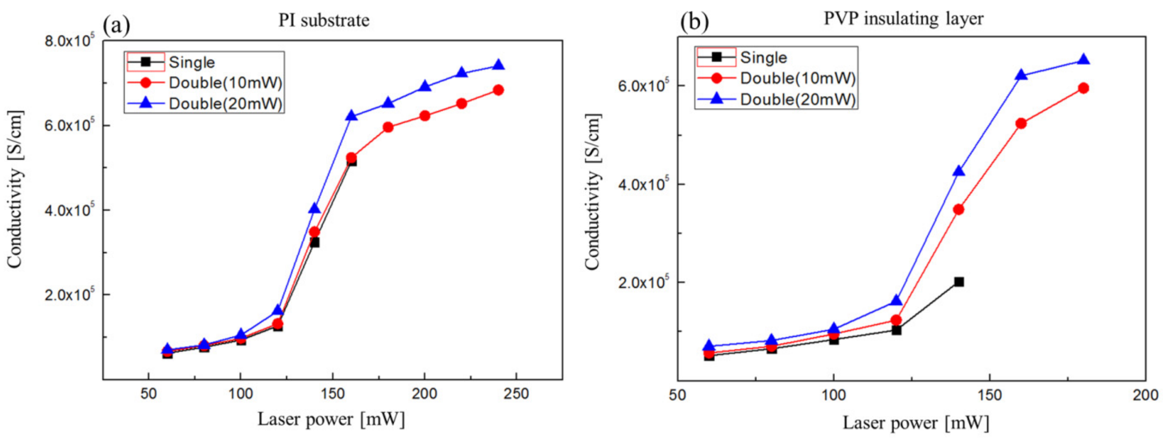

Figure S3. Electric conductivity versus laser power on (a) PI substrate and (b) PVP insulating layer.
